# Supplementary material for: A pre – post quasi-experimental study of team-based learning effectiveness for Vietnamese nursing students
Source: PLoS One. 2025 May 28;20(5):e0323656. doi: 10.1371/journal.pone.0323656 (PMC12118889; doi:10.1371/journal.pone.0323656)
Supplement: Attachment — Submitted filename: review.docx [file pone.0323656.s002.docx]

In the last paragraph of the introduction, we should see the study objective. Please clearly mention the objectives in the text.

The process for translation and validation of tools are unclear.

Did the normality condition was checked for all tools? What was the statistical parameters for checking the normality?
