# Supplementary material for: A pre – post quasi-experimental study of team-based learning effectiveness for Vietnamese nursing students
Source: PLoS One. 2025 May 28;20(5):e0323656. doi: 10.1371/journal.pone.0323656 (PMC12118889; doi:10.1371/journal.pone.0323656)
Supplement: Attachment — Submitted filename: Response to Reviewers.docx [file pone.0323656.s004.docx]

|  | **Requirements** | **Comments from reviewers** | **Edit** |
| --- | --- | --- | --- |
| 1 | Please ensure that your manuscript meets PLOS ONE's style requirements, including those for file naming |  | We have edited as requested. |
| 2 | [Please remove all personal information, ensure that the data shared are in accordance with participant consent, and re-upload a fully anonymized data set.](http://track.editorialmanager.com/CL0/http:%2F%2Fwww.bmj.com%2Fcontent%2F340%2Fbmj.c181.long./1/010f019582fb966a-6e62cefc-4070-4e23-907b-7def52a07cc8-000000/QR7uoenvub2jy2ss9N7xvWRc7otiiQVMMgCB8Z2lEpI=201) |  | We have edited as requested. |
| 3 | Please review your reference list to ensure that it is complete and correct. | Reviewer 1  Introduction  1- improve the introduction with newer study 2- firs paragraph of introduction is too long. 3- highlight importance of using TBL is good, but you have not integration in introduction. 4- the end of introduction is better you note your goal of study.  Method:  1- note how many students were excluded and why? How many was Research population? 2- describe your course: how many section or hours? About course class how many classes and how many students were in each class? | - We have updated the latest research. - We have shortened and ensured the balance of the length of the paragraphs. - We have removed the section integrating the use of TBL. - We have added the research objectives at the end of introduction. - All 186 students participated in the study, with no students excluded - We have added the requested course description into the manuscript. |
| 4 |  | Review 2   1. In the last paragraph of the introduction, we should see the study objective. Please clearly mention the objectives in the text. 2. The process for translation and validation of tools are unclear. 3. Did the normality condition was checked for all tools? 4. What was the statistical parameters for checking the normality? | - We have added the research objectives at the end of introduction. - The translation followed the World Health Organization (WHO) guidelines, including forward translation, backward translation, and expert review to ensure linguistic and conceptual equivalence. Additionally, the content validity of the instrument has been incorporated into the manuscript. - Yes, We checked the normality condition, and the variables did not follow a normal distribution. We used the Q-Q plot method to assess the normality of the variables in this study |
